# Correspondence: Reply to ‘Oncogenic MYC persistently upregulates the molecular clock component REV-ERBα'

**DOI:** 10.1038/ncomms14918

**Published:** 2017-03-23

**Authors:** Anton Shostak, Bianca Ruppert, Axel Diernfellner, Michael Brunner

**Affiliations:** 1 Heidelberg University Biochemistry Center, Im Neuenheimer Feld 328, D-69120 Heidelberg, Germany

In two recent studies by Altman *et al*. and ourselves it was demonstrated that MYC has the potential to dampen the circadian clock by repressing the core transcription activator gene *BMAL1* (refs 1, 2). Although the main finding was reported in both studies, different mechanisms have been proposed for the MYC-dependent downregulation of the core clock genes. Altman *et al*. suggested that MYC induces REV-ERBα, which disrupts the clock by repressing *BMAL1*. We found that MYC disrupts the clock in U2OS cells through a complex with MIZ1 that directly binds to and represses *BMAL1* and *CLOCK.* Data presented here and in the accompanying correspondence strongly suggest that MYC can impact on the circadian clock through both limbs, direct repression via MIZ1 and indirect repression via induction of REV-ERBα.

Dependent on partner proteins, MYC can act as a transcription activator and repressor of a large number of genes^3^. MYC in a complex with MAX is a bHLH transcription activator that binds to E-boxes, and MYC/MAX in a complex with the transcription activator MIZ1 constitute a repressor of MIZ1 regulated genes. This repressive complex promotes cell cycle progression and growth by repressing the cyclin-dependent kinase inhibitors genes p15 and p21 (ref. 3).

The heterodimeric bHLH transcription factor BMAL1/CLOCK is a core element of the circadian clock, which activates expression of the negative regulators *PERs*, *CRYs* and *REV-ERBα/β*. PER and CRY proteins inactivate BMAL1/CLOCK while REV-ERBs are repressors of ROR-mediated transcription of *BMAL1* and *CLOCK* genes. ChIP-seq analyses indicate that MYC binds to E-boxes in *PER*, *CRY* and *REV-ERB* genes and also to MIZ1 sites in the promoters of *BMAL1* and *CLOCK* (refs 1, 2).

Altman *et al*. reported that overexpression of MYC in U2OS cells and MYCN in neuroblastoma-derived cells correlates with increased expression of REV-ERBα, and they proposed that MYC activates REV-ERBα, which then represses *BMAL1*. Based on the well-established wiring of the transcriptional network of the circadian clock there is no doubt that accumulation of sufficient amounts of REV-ERBα will repress *BMAL1*, and it has been shown experimentally that overexpressed REV-ERBα disrupts the circadian clock in mouse liver^4^.

We had shown that overexpression of MYC mutant versions that were compromised in their capacity to interact with MIZ1 but were functional as E-box-dependent activators did not substantially affect circadian oscillations in U2OS cells. This suggests a mode of MYC action different from the induction of REV-ERBα. In addition, knockdown of MIZ1 by siRNA rescued most of the MYC-induced downregulation of the circadian clock, demonstrating that MYC acts via MIZ1. Since we did not see MYC-dependent upregulation of *REV-ERBα* in U2OS cells, we concluded that MIZ1-mediated repression is the major pathway by which overexpressed MYC mutes the circadian clock in U2OS cells.

Discrepancies in the interpretation of very similar observations between Altman *et al*. and our paper are based on apparent differences in MYC-induced levels of *REV-ERBα* in U2OS cells. Altman *et al*. reported that U2OS cells overexpressing MYC displayed elevated expression of *REV-ERBα* (∼2-fold), a finding that was not observed in our study^1^,^2^. In contrast, induction of MYCN in neuroblastoma-derived cells was accompanied by a much more substantial accumulation of *REV-ERBα*.

We realized that we and Altman *et al*. used different amplicons to assess expression, and that data were normalized to different housekeepers, *GAPDH* and *B2M*, respectively. Hence, using our published samples, we reanalysed mRNA expression levels with primers used by us (same primers as Zhang *et al*.^5^) and by Altman *et al. REV-ERBα* amplicons used in both studies showed identical behaviour and when normalized to *GAPDH*, confirmed that *REV-ERBα* levels were slightly reduced in U2OS cells overexpression MYC (Fig. 1a). However, relative to *B2M*, *REV-ERBα* levels (assessed with both amplicons) were slightly elevated at 24 h and essentially unaffected at 36 h.

We confirmed this difference in normalization with independent cDNA profiles from U2OS cells overexpressing MYC (Fig. 1b). Quantification of samples using TaqMan instead of SYBR green yielded similar results (Supplementary Fig. 1a). The data demonstrate that the different reference genes (*GAPDH* versus *B2M*) used in both studies are responsible for the discrepancy in quantification of MYC-dependent expression of *REV-ERBα*. Considering that MYC is a global transcription regulator it is quite challenging to identify ideal housekeepers. Thus, due to the lack of a suitable reference it is difficult to assess small differences in *mRNA* levels triggered by overexpressed MYC in U2OS cells.

However, ultimately REV-ERBα protein rather than RNA will impact on the circadian clock. Therefore, we as well as Altman *et al*. (see correspondence) analysed REV-ERBα protein. We tested two independent U2OS clones (#C8 and #10) harbouring inducible MYC. In clone #C8, which was used in our published study, REV-ERBα protein levels decreased upon MYC induction, whereas in clone #10 the levels increased slightly (Supplementary Fig. 1b). A more detailed circadian profile of clone #10 revealed that expression levels of REV-ERBα were generally slightly higher when MYC was induced, with the exception of the 48 h timepoint, which corresponds to the circadian peak of REV-ERBα (Fig. 1c). In average, REV-ERBα levels were about 1.2-fold higher in U2OS cells overexpressing MYC (Fig. 2a). This raises the question whether this moderate increase in REV-ERBα levels is sufficient to account for the MYC-induced disruption of the circadian clock.

Interestingly, MYC-dependent downregulation of BMAL1 and CLOCK was observed in both cell lines (clones #C8 and #10) independent of whether REV-ERBα levels were slightly reduced (#C8) or elevated (#10). This suggests that the downregulation of BMAL1 and CLOCK did not correlate with REV-ERBα levels.

We then analysed the relative repressive potential of MYC-induced REV-ERBα in comparison to the MYC/MIZ1-mediated direct repression of *BMAL1* and *CLOCK*. When *REV-ERBα* was downregulated with specific siRNA^1^, REV-ERBα protein was efficiently depleted (Fig. 2a). As expected, absence of REV-ERBα was accompanied by elevated expression of BMAL1 and CLOCK, demonstrating the repressive capacity of REV-ERBα in U2OS cells. However, induction MYC resulted in a two- to threefold reduction of BMAL1 and CLOCK protein levels in absence and presence of REV-ERBα (Fig. 2a), indicating that MYC can repress *BMAL1* and *CLOCK* by a pathway independent of REV-ERBα.

REV-ERBs and RORs regulate expression of *BMAL1* via ROR elements (ROREs). Mutation of both ROREs in a *Bmal1-luc* reporter disrupts its activation by RORs and its inhibition by REV-ERBs, and thus leads to an arrhythmic luciferase expression profile^6^,^7^. To assess whether downregulation of *Bmal1-luc* by overexpressed MYC is dependent on intact ROREs we constructed a *Bmal1-luc* reporter lacking both ROREs (*Bmal1*-Δ*RORE1,2-luc*). U2OS cells transfected with *Bmal1*-Δ*RORE1,2-luc* expressed luciferase in arrhythmic fashion (Fig. 2b). However, *Bmal1*-Δ*RORE1,2-luc* was still repressed upon induction of MYC indicating a REV-ERB-independent mechanism of inhibition. Nonetheless, the MYC-induced repression of *Bmal1*-Δ*RORE1,2-luc* was slightly weaker as compared to the wild-type *Bmal1-luc* reporter (60% versus 80% of repression at the first peak, respectively) suggesting that REV-ERBs contribute to the repression in addition to MYC/MIZ1. Hence, direct repression of *BMAL1* and *CLOCK* by MYC/MIZ1 appears to be the dominant pathway by which MYC disrupts the circadian clock in U2OS cells.

However, in comparison to the U2OS system, Altman *et al*. observed a much stronger upregulation of REV-ERBα induced by MYCN in neuroblastoma-derived cell lines (accompanying correspondence). Under these conditions REV-ERBα is likely to be the dominant repressor disrupting the circadian clock.

Taken together, both papers and this correspondence show that MYC can in principle impinge via two pathways on the circadian clock (Fig. 2c). On one hand, MYC is an E-box-dependent activator with the potential to indirectly affect the clock by inducing REV-ERBα/β, which are repressors of *BMAL1* and *CLOCK*. On the other hand, MYC, in a complex with MIZ1, is a direct repressor of *BMAL1* and *CLOCK*. Whether these pathways are effective and which of both pathways is prevalent may depend on the cell type and conditions. It is tempting to speculate that the MIZ1- and REV-ERB-dependent pathways could be regulated independently and thus cooperatively modulate circadian gene expression according to different developmental states, growth conditions and metabolic cues.

## Methods

### Cell culture and transfections

U2OS t-rex tetO-MYC cells^1^ were maintained in DMEM supplemented with 10% FBS, 1x PenStrep, 50 μg ml^−1^ hygromycin, and 50 μg ml^−1^ zeocin (InvivoGen). All reagents for the cell culture were purchased from Life Technologies unless indicated differently. Twenty-four hours before synchronization, U2OS cells were transfected with siRNAs^1^ using lipofectamine RNAiMAX reagent according to the manufacturer's protocol. For luciferase recordings, U2OS cells were transfected with reporter constructs using Xfect (Clontech) and next day, cells were synchronized with 1 μM dexamethasone for 20 min and washed with PBS. After addition of warm luminescene medium (DMEM w/o Phenolred supplemented with 10% FBS, 25 mM Hepes, 1x PenStrep, and 0.125 μM luciferin (BioSynth), 10 ng ml^−1^ doxycycline) the plate was sealed and bioluminescence was recorded at 37 °C with an EnSpire Reader (Perkin Elmer).

### Gene expression analysis

Total RNA was isolated using TriFaster (GeneON) and cDNA was synthesized with Maxima First Strand cDNA Synthesis Kit (Thermo Scientific). qPCR was performed using Green-Dye-Mastermix (Steinbrenner), TaqMan Gene Expression Master Mix (Applied Biosystems) and LightCycler 480 (Roche), and relative gene expression was quantified using a ΔΔCt method with respective reference gene (*GAPDH* or *B2M*). SYBR green primers were published previously^2^,^5^. TaqMan primer sequences are listed in Supplementary Table 1.

### Western blotting

Protein lysates were prepared from synchronized U2OS t-rex tetO-MYC cells and blotted on nitrocellulose membranes. Membranes were incubated in 5% milk TBS at 4 °C overnight supplemented with anti-BMAL1 (ref. 1) (1:750), anti-CLOCK (ref. 1) (1:500), anti-Tubulin (1:1,000, WA3), anti-MYC (1:400, N-262, SantaCruz) and anti-REV-ERBα (1:1,000, 13418S, Cell Signaling) antibodies. Proteins were quantified using ImageJ by normalizing to Tubulin. Uncropped blots are shown in Supplementary Fig. 2.

### Data availability

The data supporting the findings of this study are available within the article and its Supplementary Information files, or from the authors on request.

## Additional information

**How to cite this article:** Shostak, A. *et al*. Correspondence: Reply to ‘Oncogenic MYC persistently upregulates the molecular clock component REV-ERBα'. *Nat. Commun.* **8,** 14918 doi: 10.1038/ncomms14918 (2017).

**Publisher's note**: Springer Nature remains neutral with regard to jurisdictional claims in published maps and institutional affiliations.

## Supplementary Material

Supplementary Information Supplementary Figures and Supplementary Table

## Figures and Tables

**Figure 1:**
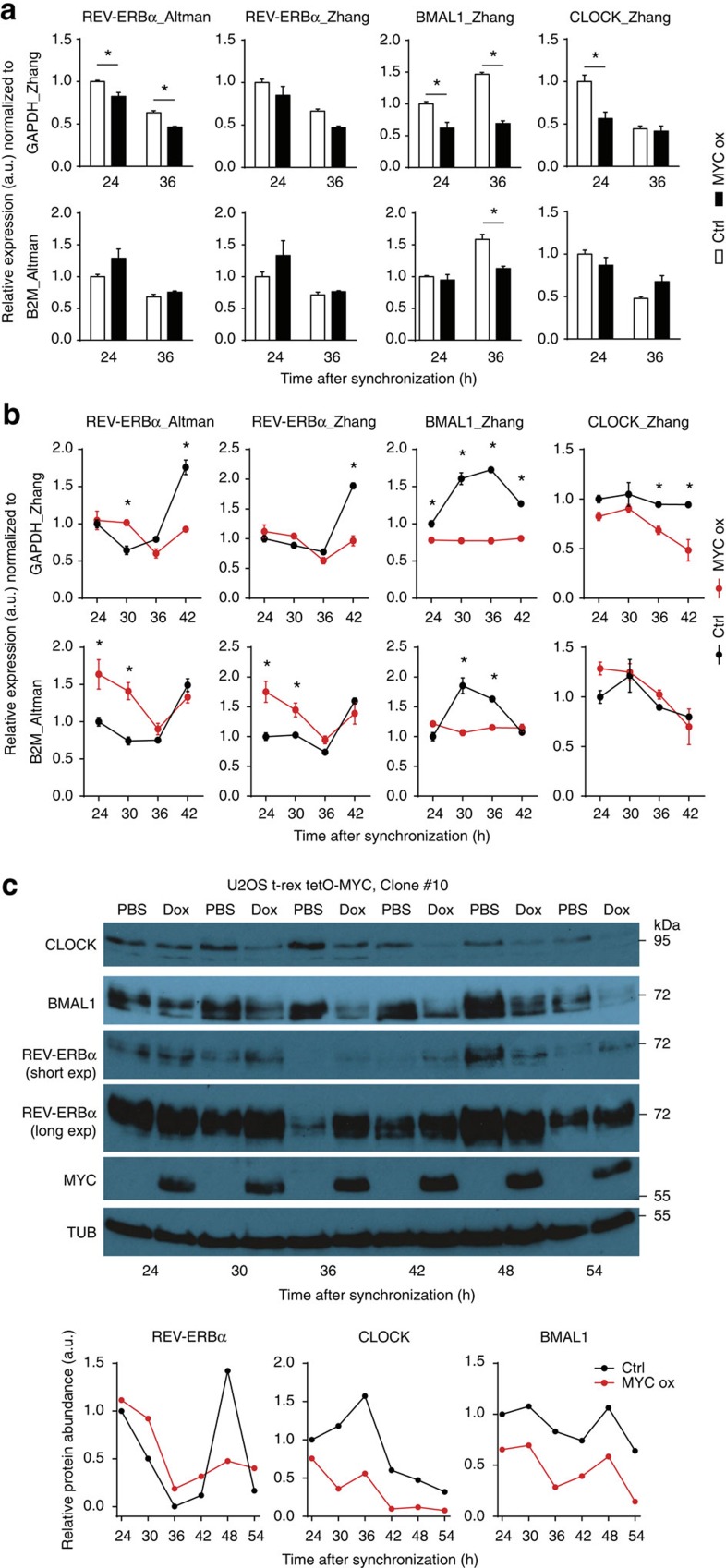
Overexpression of MYC moderately increases REV-ERBα in U2OS cells. (**a**) qPCR reevaluation of RNA expression data from Shostak *et al*.^1^ at two circadian time points using primers from Zhang *et al*.^5^ and Altman *et al*.^2^. Data were normalized to *GAPDH* (upper row) or *B2M* (lower row) by ΔΔCt (*n*=3). (**b**) qPCR analysis of circadian expression profiles of indicated transcripts in synchronized U2OS t-rex tetO-MYC cells normalized to *GAPDH* (upper row) or *B2M* (lower row) (*n*=3). qPCR products were detected with SYBR Green, corresponding quantification with TaqMan probes is shown in Supplementary Fig. 1a. (**c**) Western blot analysis (upper panel) and densitometric protein quantification (lower panel) of indicated proteins in synchronized U2OS t-rex tetO-MYC cells in presence (Dox) and absence (PBS) of overexpressed MYC (*n*=1). Data are presented as mean±s.e.m. *P<0.05; two-way ANOVA with Bonferroni post-test.

**Figure 2:**
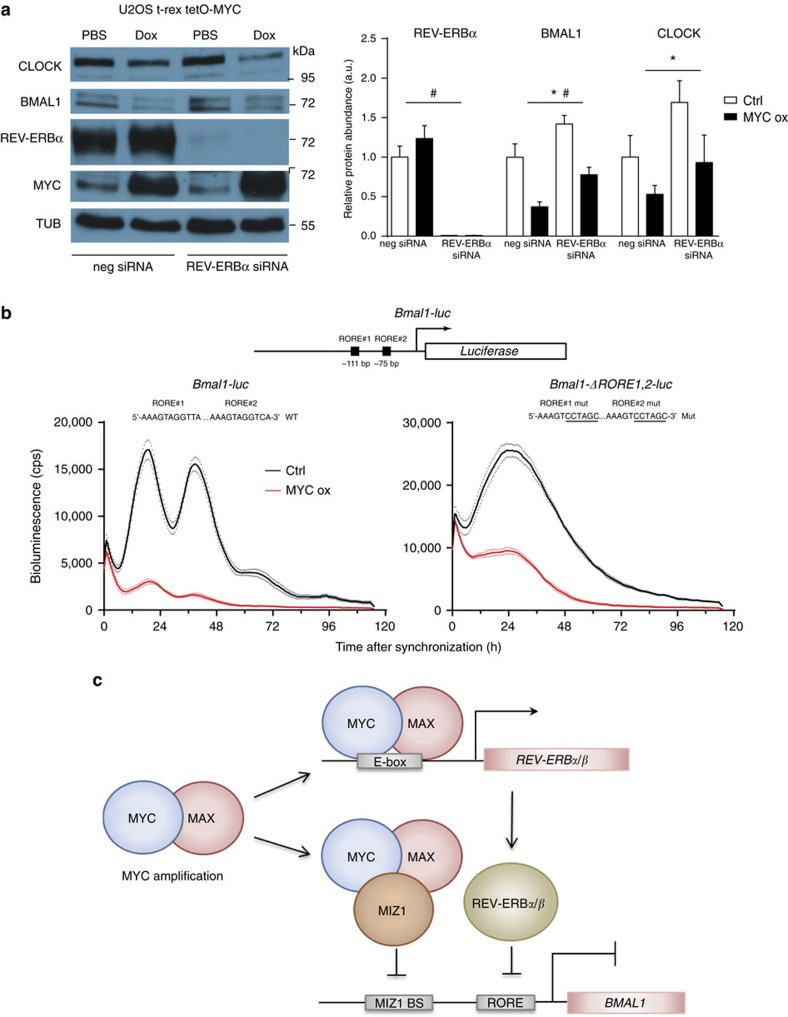
MYC represses BMAL1 and CLOCK in absence of REV-ERBα. (**a**) MYC-dependent reduction of BMAL1 and CLOCK levels in presence and absence of REV-ERBα. U2OS t-rex tetO-MYC cells were transfected with *REV-ERBα* or negative siRNAs as indicated. After 24 h cells were treated with doxycycline to induce MYC (MYC ox) or with PBS for control (Ctrl). Western blot analysis (left) and densitometric protein quantification (right) (*n*=3) are shown. *P<0.05 for Ctrl versus MYC ox; ^#^*P*<0.05 for neg siRNA versus REV-ERBα siRNA by two-way ANOVA. (**b**) MYC-dependent repression of *Bmal1* promoters with and without ROREs. Schematic of the *Bmal1-luc* construct showing the location of ROREs. Sequence changes inactivating ROREs^6^,^7^ are underlined (right panel). U2OS t-rex tetO-MYC cells transiently transfected with *Bmal1-luc* (left) or *Bmal1-ΔRORE1,2-luc* (right) were treated with doxycycline (MYC ox) or PBS (Ctrl) as indicated and, bioluminescence traces were recorded (*n*=3). Data are presented as mean±s.e.m. (**c**) Schematic of MYC-dependent direct and indirect repression of BMAL1.
