# Supplementary material for: Correspondence: Reply to ‘Oncogenic MYC persistently upregulates the molecular clock component REV-ERBα'
Source: Nat Commun. 2017 Mar 23;8:14918. doi: 10.1038/ncomms14918 (PMC5376643; doi:10.1038/ncomms14918)
Supplement: Supplementary Information — Supplementary Figures and Supplementary Table [file ncomms14918-s1.pdf]

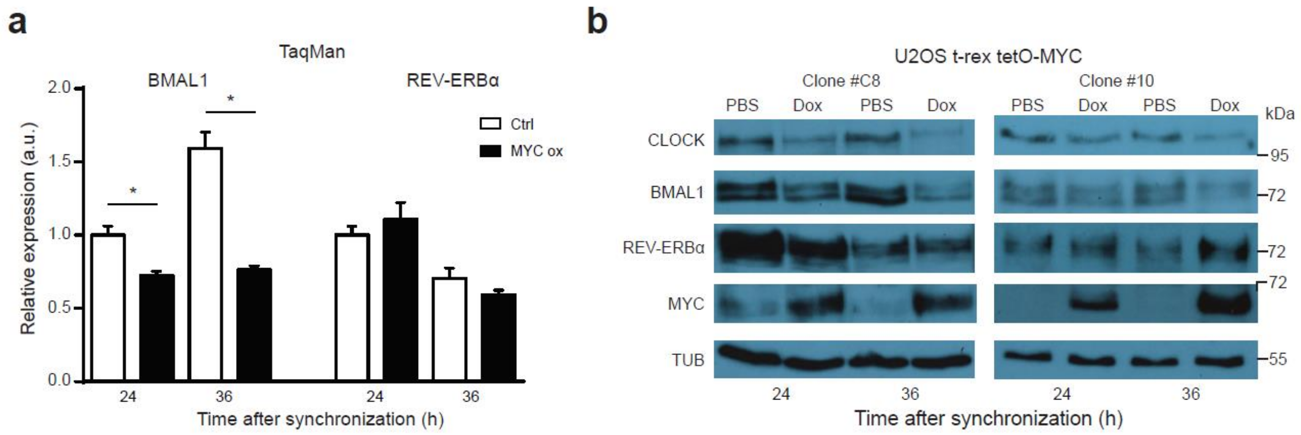

Supplementary Figure 1. Effects of MYC overexpression on REV-ERBα levels in U2OS cells

(a) TaqMan qPCR analysis of *BMAL1* and *REV-ERBα* transcripts normalized to *GAPDH* from Figure 1b at 24 and 36 hours after synchronization (n=3). **(b)** Overexpressed MYC correlates with reduced expression of BMAL1 and CLOCK, while REV-ERBα levels are different in two cell clones. Western blot analysis of indicated proteins in synchronized U2OS t-rex tetO-MYC cells treated with doxycycline to induce MYC (Dox) or with PBS for control. Data are presented as mean ± SEM. * $P < 0.05$; two-way ANOVA with Bonferroni post-test.

for Figure 1c

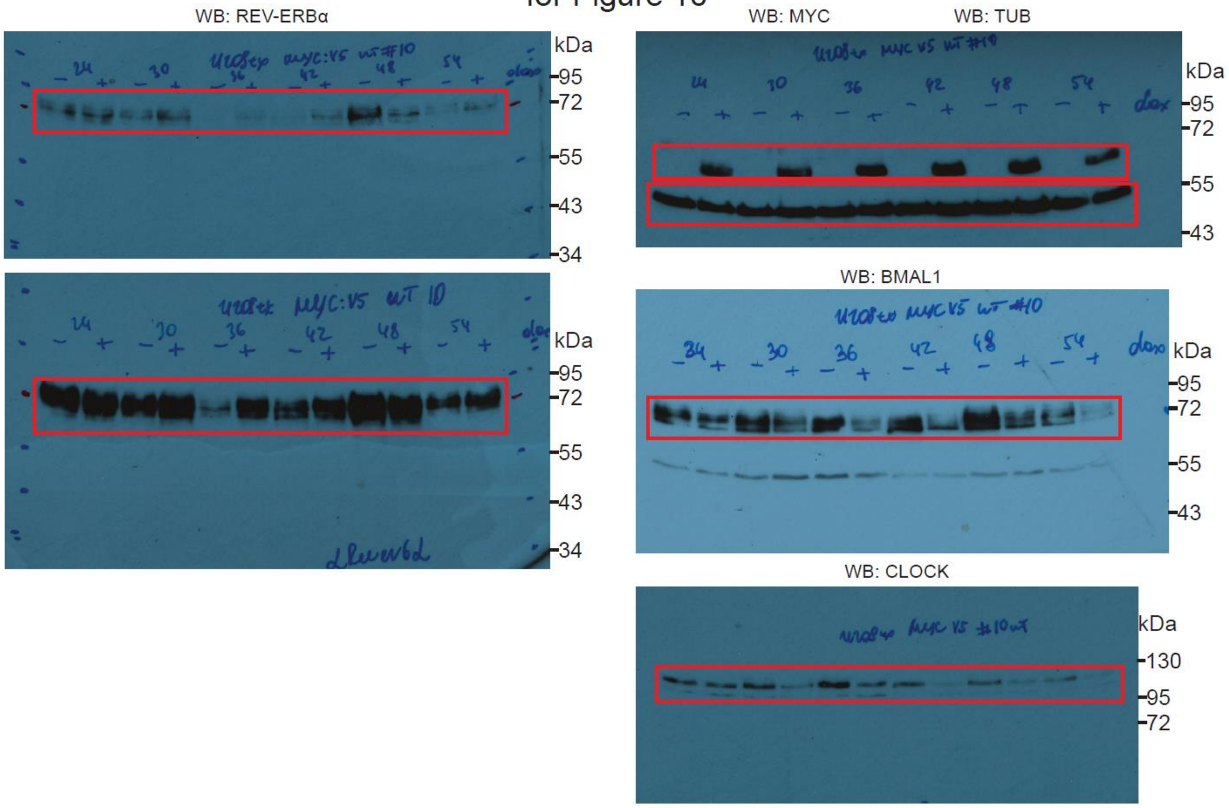

for Figure 2a

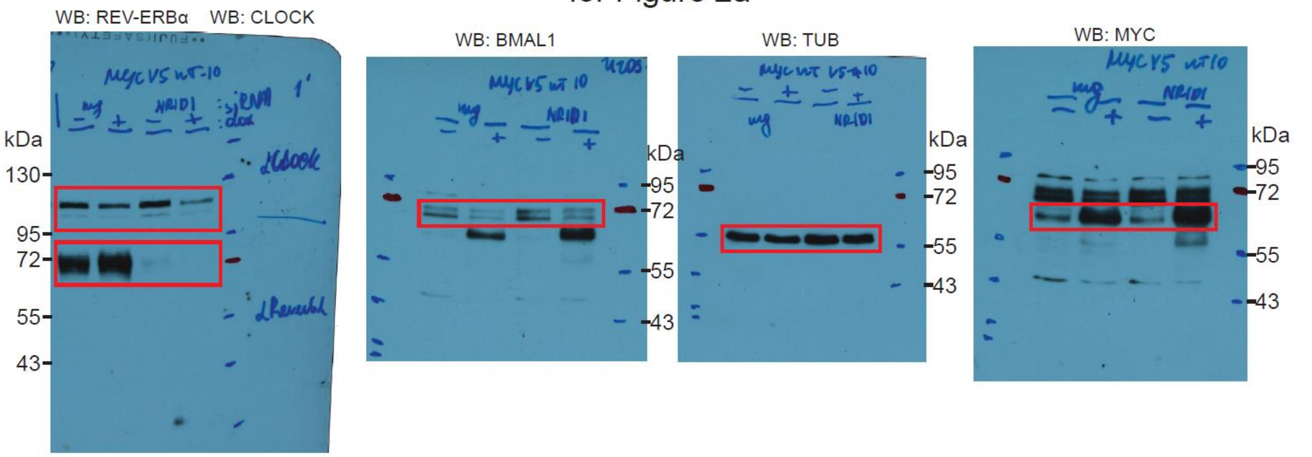

Supplementary Figure 2. Full scans of Western blots shown in main figures

Cropped areas are marked by red color

Supplementary Table 1. TaqMan primer sequences

Gene	Primer/probe sequence
hGAPDH_F	catcaatggaaatcccatca
hGAPDH_R	gactccacgacgtactcagc
hGAPDH_probe	6-FAM-tccaggagcgagatccctcca-TARMA
hBMAL1_F	ttggacgactgcattctcat
hBMAL1_R	aatagctgttgccctctggt
hBMAL1_probe	6-FAM-tccatctatcgcgtgccgaga-TARMA
hREV-ERBα_F	acctttgaggtgctgatgg
hREV-ERBα_R	ggaacatcactgtctggtcct
hREV-ERBα_probe	6-FAM-tgcgctttgcttcgttgttcaa-TARMA
